# Tumor-related molecular determinants of neurocognitive deficits in patients with diffuse glioma

**DOI:** 10.1093/neuonc/noac036

**Published:** 2022-02-11

**Authors:** Emma van Kessel, Sharon Berendsen, Anniek E Baumfalk, Hema Venugopal, Eva A Krijnen, Wim G M Spliet, Wim van Hecke, Fabrizio Giuliani, Tatjana Seute, Martine J E van Zandvoort, Tom J Snijders, Pierre A Robe

**Affiliations:** University Medical Center Utrecht, UMC Utrecht Brain Center, Department of Neurology & Neurosurgery, Utrecht, The Netherlands; University Medical Center Utrecht, UMC Utrecht Brain Center, Department of Neurology & Neurosurgery, Utrecht, The Netherlands; University Medical Center Utrecht, UMC Utrecht Brain Center, Department of Neurology & Neurosurgery, Utrecht, The Netherlands; University Medical Center Utrecht, UMC Utrecht Brain Center, Department of Neurology & Neurosurgery, Utrecht, The Netherlands; University Medical Center Utrecht, UMC Utrecht Brain Center, Department of Neurology & Neurosurgery, Utrecht, The Netherlands; University Medical Center Utrecht, Department of Pathology, Utrecht, The Netherlands; University Medical Center Utrecht, Department of Pathology, Utrecht, The Netherlands; University Medical Center Utrecht, UMC Utrecht Brain Center, Department of Neurology & Neurosurgery, Utrecht, The Netherlands; University Medical Center Utrecht, UMC Utrecht Brain Center, Department of Neurology & Neurosurgery, Utrecht, The Netherlands; University Medical Center Utrecht, UMC Utrecht Brain Center, Department of Neurology & Neurosurgery, Utrecht, The Netherlands; Helmholtz Institute, Utrecht University, Utrecht, The Netherlands; University Medical Center Utrecht, UMC Utrecht Brain Center, Department of Neurology & Neurosurgery, Utrecht, The Netherlands; University Medical Center Utrecht, UMC Utrecht Brain Center, Department of Neurology & Neurosurgery, Utrecht, The Netherlands; Liège University Hospital, Department of Human Genetics, Liège, Belgium

**Keywords:** cognitive functioning, diffuse glioma, oncobiological characteristics

## Abstract

**Background:**

Cognitive impairment is a common and debilitating symptom in patients with diffuse glioma, and is the result of multiple factors. We hypothesized that molecular tumor characteristics influence neurocognitive functioning (NCF), and aimed to identify tumor-related markers of NCF in diffuse glioma patients.

**Methods:**

We examined the relation between cognitive performance (executive function, memory, and psychomotor speed) and intratumoral expression levels of molecular markers in treatment-naive patients with diffuse glioma. We performed a single-center study in a consecutive cohort, through a two-step design: (1) hypothesis-free differential expression and gene set enrichment analysis to identify candidate oncogenetic markers for cognitive impairment. Nineteen molecular markers of interest were derived from this set of genes, as well as from prior knowledge; (2) correlation of cognitive performance to intratumoral expression levels of these nineteen molecular markers, measured with immunohistochemistry.

**Results:**

From 708 included patients with immunohistochemical data, we performed an in-depth analysis of neuropsychological data in 197, and differential expression analysis in 65 patients. After correcting for tumor volume and location, we found significant associations between expression levels of CD3 and IDH-1 and psychomotor speed; between IDH-1, ATRX, NLGN3, BDNF, CK2Beta, EAAT1, GAT-3, SRF, and memory performance; and between IDH-1, P-STAT5b, NLGN3, CK2Beta, and executive functioning. P-STAT5b, CD163, CD3, and Semaphorin-3A were independently associated after further correction for histopathological grade.

**Conclusion:**

Molecular characteristics of glioma can be independent determinants of patients’ cognitive functioning. This suggests that besides tumor volume, location, and histological grade, variations in glioma biology influence cognitive performance through mechanisms that include perturbation of neuronal communication. These results pave the way towards targeted cognition improving therapies in neuro-oncology.

Key Points

- Cognitive impairment in diffuse glioma patients is the result of multiple factors
- Several oncobiological characteristics of gliomas are determinants of cognitive functioning

**Importance of the Study**

Our findings confirm that variations in glioma biology correlate with cognitive performance. We found several new biomolecular characteristics that suggest a metabolic perturbation of neuronal and non-neuronal signaling by glial tumors.

These findings improve our understanding of the pathophysiology of cognitive deficits in glioma and pave the way towards follow-up studies that (help) define the clinical risk of developing cognitive sequelae among glioma patients as well as developing treatments against them.

Diffuse glioma (WHO grade II-IV) is a group of primary brain tumors with a variable prognosis, but an invariably fatal outcome. Treatment is therefore based on a multidisciplinary palliative approach with the aim to maintain health-related quality of life (HRQoL), establish tumor inhibition, and prolong life.

Approximately 60% of the patients with diffuse glioma suffer from neurocognitive impairment with involvement of one or multiple cognitive domains, even before the initiation of tumor-directed treatment.^1,2^ Moreover, patients with incidentally discovered low-grade glioma commonly present with an early neurocognitive decline in absence of any other symptoms.^1^ Neurocognitive dysfunction strongly limits HRQoL in glioma patients and their families.^3^

Tumors exert local mass-related compression, dislocation, or ischemia of healthy neural tissue that is the basis of the cognitive dysfunction of glioma patients.^4^ Glioma patients however tend to show a broad range of cognitive impairments including impaired memory, executive functions, psychomotor function, attention, and information processing.^2^ Moreover, only weak correlation between tumor localization and cognitive impairment has been described.^5,6^ This calls for other hypotheses as underlying explanation for these broad range of deficits.

First, the hodotopic functional organization of the brain allows tumors in different locations and sizes to alter the brain connectome that underlies a specific cognitive function in a similar fashion.^7^ Accordingly, neuroimaging studies have demonstrated widespread disturbances and reduced organizational efficiency in functional neural networks in glioma patients which correlate to cognitive status.^8–10^

Second, individual background of the patients both at the level of education and socio-economical status as well biological can modulate the cognitive impact of tumors. Regarding the latter, single nucleotide polymorphisms genotypes of APOE, NOS1, and IL16, ABCC, POLE, or ERCC4 correlate with different susceptibility to neurocognitive deficits.^11–13^

Third, besides its size and location,^14^ the metabolism of a tumor can alter neural function. The favorable cognitive profile of patients with an isocitrate dehydrogenase (IDH) 1 or 2 gene- mutated glioma is an example of this.^15^ Mutations in IDH 1 and 2 occur in the vast majority of low-grade gliomas and in secondary high-grade gliomas. These mutations alter the function of the Krebs cycle enzymes, causing them to produce, instead of alpha-ketoglutarate, 2-hydroxyglutarate, a possible oncometabolite that regulates DNA methylation, HIF-1alpha signaling, and mitochondrial function. This oncometabolite is also released in the extracellular space where it can alter normal cells in the tumor microenvironment, including the function of tumor-infiltrating lymphocytes^16^ and neurons.^17^ In addition, IDH-mutated tumors grow slower than their wild-type counterparts and may allow additional plasticity of the surrounding nervous tissue during tumor development. This results in a complex interrelationship between patients’ neurocognitive function, tumor growth velocity, and the presence or absence of an IDH-mutation.^15^

Likewise, diffuse gliomas release various extracellular effectors such as glutamate (an excitotoxic neurotransmitter)^18^ or growth factors like BDNF, a potentiator of long term memory at the synaptic level.^19^ They form hybride tumor-astrocyte junctions that allow the passage of tumor metabolites, Calcium ions, and miRNA towards these cells.^20^ Such mechanisms alter astrocytic networks and neuronal synaptic organization^21–23^ and could in theory account for cognitive function disturbances. Better insight on the molecular basis of neurocognitive dysfunction in diffuse glioma could aid in predicting the cognitive effects of treatment for individual patients, as well as in identification of targets for cognition-directed therapy.

In this study, we aimed to identify metabolic tumor-related markers of neurocognitive functioning (NCF) in a single-center, prospectively collected patient cohort of diffuse glioma. First, we performed differential expression and gene set enrichment (GSEA) analyses of a subset of our tumor samples to identify candidate oncogenetic markers for cognitive impairment in a hypothesis-free manner. Eleven molecular markers of interest were derived from this set of genes. Eight additional factors were selected based on prior knowledge/literature (IDH-1, p53, Cx30, p-ROCK1, GAT-3, LRP-4, BDNF, and ATRX). In the second step of analysis, we correlated cognitive performance (executive function, memory, and psychomotor speed) to tumoral expression levels of these 19 molecular markers in our complete cohort of treatment-naive patients with a diffuse glioma.

## Materials and Methods

### Ethics Statement

This study was conducted following approval by the local ethical committee and institutional review board, and after obtaining written informed consent (17-384).

### Patient Selection and Study Design

A retrospective cohort study of a consecutive cohort of 793 adult patients with proven diffuse glioma (WHO grade II–IV) at the UMCU was conducted. The participants all underwent first surgical tumor resection under either awake conditions (*n* = 197) or general anesthesia (*n* = 596) in the period of January 2010 to January 2017. The selection of included patients is showed in a flowchart in Supplementary Figure 1. Inclusion-criteria were a minimum age of 18 years, a first diagnosis of diffuse glioma (WHO grade II–IV) in agreement with the WHO 2016 criteria and (first) surgical tumor resection. Exclusion criteria were (1) any anti-tumor treatment (surgery, radiotherapy, or chemotherapy) given before neuropsychological assessment (NPA), (2) unavailable or not assessable tissue for tissue microarray (TMA) analysis, (3) excessive amount of missing NPA data within a cognitive domain. Data were considered complete if more than 50% of tasks within one domain were performed.

### Baseline Characteristics and Neuropsychological Tests

Patient characteristics were retrospectively retrieved from the electronic patient files. Those included age, gender, Karnofsky Performance Score (KPS), WHO 2016 classification of gliomas, and tumor location and volume. We categorized tumor location according to the involvement of different brain lobes, with most tumors involving more than one lobe. This classification of location (lobar involvement) offers an unbiased presentation of the brain regions that may be influenced by the glioma. In addition, we categorized location into single, overall (mostly multilobar) locations per patient, as this may offer a better reflection of the involvement of brain structures, and associated networks, per patient. For analyses, we used categorization per lobe.

All NPA data was prospectively collected as part of routine clinical care. The tests that were used for NPA remained the same during this period of time (from 2010 to 2017).

The neuropsychological instruments that were used as part of our preoperative routine clinical care are listed in Supplementary Table 1. These tests are internationally widely used, standardized psychometric instruments for assessing neurocognitive deficits in the major neurocognitive domains. All tests have normative data that take into account age and, when appropriate, educational level and gender.

To determine the percentage of impaired patients at domain level, we counted the number of *individual* patients with an impaired performance per domain. A patient was considered impaired in a given domain if the patient performed below –2 SD on *any* of the administered tests within that domain. In order to identify both severe and more subtle abnormalities in NCF, we used different thresholds of –1 SD and –2 SD.

### Step 1: mRNA Expression Analysis and Pathway Analysis

Not the entire cohort had fresh frozen tumor samples available for analyses, we therefore, performed analyses on a representative subgroup of 65 de novo diffuse gliomas. RNA was extracted from sixty-five fresh-frozen surgical samples which were prospectively collected between 2010 and 2015. Quality control and differential gene expression analyses were performed with RStudio (v1.1.463). Robust Multi-array Average (RMA) normalization was applied. We analyzed differential expression with the “limma” package with correction for batch, gender, and age. Gene set enrichment analyses (GSEA’s) were performed with software provided from the broad institute as reported previously.^24^

### Step 2: Tissue Microarray and Immunohistochemistry

Archival formalin fixed paraffin embedded (FFPE) tumor tissues from 708 glioma patients operated in the UMCU between 2010 and 2017 were included in tissue microarrays (TMA’s). Tissue material contained biopsies and (sub) total resections. Tumor classification (type and grading) was performed by senior clinical neuropathologists as part of routine care and was based on the 2007 CNS tumor classification criteria. For our current analysis, all tumor samples dating before 2016 were re-classified according to WHO 2016 criteria.

TMA construction and immunostaining were performed at the pathology department of the UMCU. For each patient, three representative tumor zones for core sampling were delineated and marked on the original haematoxylin and eosin (HE)-stained samples by two neuropathologists (WvH and WGMS). Antibody titration was performed on control tissues by means of a dilution series until the staining intensities corresponded with those depicted in the antibody supplier manual. The antibodies and titration that were used are provided in Supplementary Table 2.

Protein expression assessment was blinded to clinical data and performed by means of light microscopy (magnifications 25x, 50x, 100x, 200x). The tumor samples were scored by medical students (HV, EAK, AEB) and junior clinical scientists (EvK, SB), who all underwent training by an experienced neuropathologist (WvH and WGMS).

More information regarding materials and methods can be found in Supplementary Table 3.

### Statistical Analysis

Statistical analyses were performed with IBM SPSS statistics 26.0. for Mac (SPSS Inc., Chicago, IL, USA) and Rstudio (v1.1.463). *P*-values <.05 were considered statistically significant.

Impairments or deficits (–2 SD or –1SD) in executive functioning, memory, and psychomotor speed were chosen as primary outcome measures.

All baseline characteristics were expressed as frequencies (n, %), means and medians.

#### mRNA expression analysis and pathway analysis.—

Differences in RNA expression between patients with and without cognitive impairments for three different domains (executive functioning, memory, and psychomotor speed) were analyzed by empirical bayesian statistics in the ‘limma’package in Rstudio (v1.1.463). To control for inflation of type I error by multiple testing, *P*-values were adjusted by default false-discovery rate (FDR) procedure. Adjusted *P*-values <.05 were considered significant. All results were corrected for batch, gender, and age.

GSEA was performed on the web-based GSEA server provided by the Broad Institute. Phenotypes were patients with and without cognitive impairments for the different cognitive domains. GSEA evaluates a query microarray data set by using a collection of different types of gene sets; cytogenetic, functional, Regulatory-motif sets, and neighborhood sets. Details about the consistence of these sets can be found on the website of the Broad Institute and in previous reported literature.^24^ Pathways with FDR ≦ 0.25 were considered significant. From these pathways, we selected antibodies and tests for step 2 based on (a) findings from GSEA/single-gene analysis; (b) availability of IHC antibodies; (c) final decisions from group discussions.

#### Tissue microarray and immunohistochemistry.—

To get in touch with the data, first univariable logistic regression analyses (number 1) were applied to determine the correlation between molecular markers and cognitive performance by means of Chi-Square-test. Then we performed pre-planned multivariable analyses for all different proteins, for both thresholds, for all domains, corrected for tumor grade (WHO 2007), tumor-volume, and location (number 2). For this specific preplanned analysis, the covariates were selected in advance, in line with the etiological design of our study, rather than selecting the covariates on the basis of univariable analyses.

To investigate more exploratively whether we missed associations because they were suppressed by adjusting for grade (ie overcorrection), we performed another multivariable analysis (number 3). We selected markers based on univariable analyses (*P* < .1) and then corrected only for volume and location of the tumor in multivariable regression. Again, for all three domains and for both thresholds of cognitive performance (–2 SD or –1 SD). We selected markers in univariable analysis with a domain score of –2 as outcome; we then investigated the selected markers multivariably for both thresholds of –1 and –2. We used both thresholds in multivariable analysis to gain insight into possible underlying pathways that may be related to tumor-grade per different domain, at different levels of symptoms severity (ie different SD-thresholds). We did not repeat these analyses for all markers to avoid overtesting.

Finally, since histomolecular tumor type can have a major influence on our results beyond histological grading, we performed additional analyses (number 4). We performed the abovementioned analyses (number 1 and 3) for the subgroups of IDH-mutated and IDH-wildtype gliomas separately. It was not possible to perform analysis number 2 stratified by IDH-mutation, as WHO 2007 grade is highly collinear with IDH-mutation. In Supplementary Table 4, all the different types of analyses we performed are listed.

## Results

### Clinical Characteristics

In total 197 eligible patients, who underwent awake surgery between 2010 and 2017, were included (see Supplementary Figure 1). In total 596 patients were included in the cohort operated under general anesthesia of which four patients had to be excluded because of missing values for all molecular markers. Descriptive characteristics from the entire awake operated cohort and the patients with fresh-frozen tumor samples are presented in Table 1 and in Supplementary Table 5 respectively. Age ranged from 19 to 82 years. With respect to the WHO tumor classification, 48.2% were glioblastoma (IDH-WT and IDH-M), 21.8% were grade II/III oligodendroglioma with 1p/19q-codeletion, 23.9% were grade II/III astrocytoma with IDH-mutation, and only 6.1% were grade II/III astrocytoma IDH-WT. For the domains executive functioning and memory, only 2.0% of data was missing. The reason for having insufficient cognitive data was often emergency surgery in case of rapid clinical decline, All other variables had missing values between 1–2%, except WHO-2016 classification (11%) and KPS (5%).

**Table 1:** Baseline Characteristics of all Eligible Patients, Who Underwent Awake Surgery Between 2010 and 2017

	*N* (%)
Overall	197
Sex (Female)	69 (35.0)
Age at first surgery (mean (SD))	51.7 (14.7)
Domain Memory impaired	36 (18.3)
Domain Executive functioning impaired	51 (25.9)
Domain Psychomotor Speed impaired	38 (20.2)
Karnofsky Performance Score (median [IQR])	90.00[80.00,90.00]
Volume (cm^3^) (median [IQR])	54.91 [23.15, 101.56]
**WHO 2016 classification**	
“ II + III astro IDH-M”	47 (23.9)
“ II + III oligo IDH 1p19q codeletion”	43 (21.8)
“ II + III astro IDH WT”	12 (6.1)
“ IV GBM IDH M”	6 (3.0)
“ IV GBM IDH WT”	89 (45.2)
**WHO 2007 classification**	
Grade II	69 (35.0)
Grade III	33 (16.8)
Grade IV	95 (48.2)
**Location (measured on T2 FLAIR)** * Left frontal Left parietal Left temporal Left occipital Right frontal Right parietal Right temporal Right occipital	98 (50.3) 47 (24.1) 71 (36.4) 21 (10.8) 54 (27.7) 34 (17.4) 27 (13.8) 8 (4.1)

*Percentages add up above 100% due to involvement of multiple lobes in each patient

Cognitive impairments (Z-values ≦ –2.0) were present in 18.3% of patients for the domain memory, 25.9% for executive functioning, and 20.2%for psychomotor speed. Tumor location, classified per lobe, shows predominant involvement of frontal and temporal lobes in our cohort, mostly left-sided. Categorization of overall tumor location per patient (Supplementary Table 6) revealed that pure frontal, frontoinsular, frontotemporal (+/– insula), and multilobar (with at least frontotemporoparietal) locations were most common.

#### Gene expression analyses.—

RNA was extracted from 65 available fresh-frozen tumor samples “de novo” diffuse glioma patients of our “awake craniotomy” cohort and processed on Affymetrix HG U133 plus 2.0 arrays.

Samples were split in two groups according to the median of the neurocognitive scores in each domain. Differential gene expression was analyzed after correction for batch, gender, and age, for each cognitive domain. There was no significant (FDR ≦ 0.05) individual gene expression difference between these groups for any cognitive domain, even after correction for tumor grade.

GSEA, with an FDR discovery threshold of ≦ 0.25, however suggested the combined differential expression of genes involved in several metabolic pathways. Salient results of these analyses are provided Table 2 (analysis following correction for age, gender, and batch) and Table 3 (correction for tumor grade as well). These suggest that epilepsy-related factors (SRF, CK2Beta), miR-215-mediated translation control, transcription factor activation (STAT3), epigenetic regulation of DNA transcription (HDAC1), glutamate neurotransmitter signaling (-KEGG_AMYOTROPHIC_LATERAL_SCLEROSIS_ALS), Semaphorin/plexin signaling in neural plasticity (GO_CRANIAL_NERVE_MORPHOGENESIS and GO_PARASYMPATHETIC_NERVOUS_SYSTEM_DEVELOPMENT), and immune reaction/inflammation may correlate with neurocognition. Following inspection of the corresponding gene sets, eleven proteins were selected for further analysis on tissue microarrays as being representative for these suggested pathways. From the C2 curated gene sets, membrane transporters for excitatory amino acid EAAT1 and EAAT2; from the C3 (regulatory target gene sets) and C4 (computational gene sets) libraries: P-STAT3 (the active form of signal tranducer STAT3), Acetylated Histone 3 (H3-Ac, a major target of HDAC-1), Casein Kinase 2-beta (CK2Beta), serum response factor (SRF); C5 ontology gene sets: semaphorin3A (SEMA3) and its receptor plexin A1 (PLXNA1), and from the C7 immunologic signature gene sets: Cluster of differentiation 3 (CD3, present in T lymphocytes), Cluster of differentiation 163 (CD163, a marker of myeloid/microglial cells), and P-STAT5b the activated forms of signal transducer and activator of transcription 5.

**Table 2:** Results of Gene Set Enrichment Analysis with Correction for Batch, Age, and Gender

Pathway	Cognitive domain/phenotype	Size of gene set	NES	FDR-qvalue	CategoryGene set
CHR9P21 CHR6Q24 CHR4Q28	Executive functioning (impaired)	23 36 29	18.75 17.54 17.60	0.22 0.22 0.24	C1—positional gene sets
CHR6P12 CHR10Q26	Memory (impaired)	42 73	16.79 16.59	0.24 0.25	C1—positional gene sets
STAT3_01 SRF_C	Memory (not impaired)	16 195	-15.71 -14.49	0.11 0.21	C3—regulatory target gene sets
GNF2_MSH6 GNF2_HDAC1 GCM_CSNK2B	Memory (not impaired)	30 81 86	-15.58 -15.70 -16.01	0.19 0.19 0.21	C4—computational gene sets
GSE7509_UNSTIM_VS_TNFA_IL1B_IL6_PGE_STIM_DC_UP GSE36888_STAT5_AB_KNOCKIN_VS_WT_TCELL_IL2_TREATED_6H_DN GSE21670_IL6_VS_TGFB_AND_IL6_TREATED_STAT3_KO_CD4_TCELL_UP	Memory (not impaired)	167 172 158	-15.39 -1.61 -16.1	0.24 0.25 0.25	C7—immunologic signature gene sets
REACTOME_CGMP_EFFECTS REACTOME_NITRIC_OXIDE_STIMULATES_GUANYLATE_CYCLASE	Executive functioning (impaired)	19 24	21.65 21.77	0.01* 0.02*	C2—curated gene sets—biological pathways

NES, normalized enrichment score; FDR, false discovery rate. *=*p* < 0.05

**Table 3:** Results of Gene Set Enrichment Analysis with Correction for Batch, Age, Gender, and Grade

Pathway	Cognitive domain/phenotype	Size of gene set	NES	FDR-value	CategoryGene set
KEGG_AMYOTROPHIC_ LATERAL_SCLEROSIS_ALS REACTOME_NITRIC_OXIDE_STIMULATES_GUANYLATE_CYCLASE	Executive functioning (impaired)	53 22	0.55 0.64	<0.005* <0.005*	C2—regulatory target gene sets
TAGGTCA_MIR192_MIR215	Executive functioning (not impaired)	47	–0.51	0.19 0.19	C3—computational gene sets
GOBD_CRANIAL GOBD_PARASYMPATHETIC	Executive functioning (not impaired)	29 19	–0.73 –0.73	<0.005* <0.005*	C5—Ontology gene sets

NES, normalized enrichment score; FDR, false discovery rate.*=FDR < 0.05

#### Tissue microarray and immunohistochemistry and cognition.—

In addition to the eleven proteins selected following our GSEA analysis, conventional glioma pathology markers IDH-1, ATRX, and p53, and five other candidate markers—based on published experimental evidence on the mechanisms by which glioma cells can influence neural function in their vicinity—were selected.

Rho-associated coiled-coil-containing protein kinase 1 (p-Rock1) is a major regulator of cell invasion/migration in gliomas,^25–27^ Connexin 30 (CX30) is a major means of communication between glioma cells and can contribute to their making a network with normal astrocytes (eventually disrupting it). It also forms hemichannels capable of releasing neuroactive purine metabolites. Connexin 43(Cx43) participates in these mechanisms as well, but we have shown that Cx30 is rather an on-off expression in gliomas,^28^ whereas Cx43 can often be present and not functional in gliomas.^29^ GAT-3, the membrane transporter for the inhibitory amino acid GABA is overexpressed by glioblastoma cells^30^ and can alter the synaptic of this neurotransmitter, in a fashion similar to that of EAAT1 and 2 for the excitatory neurotransmitter glutamate. Brain-derived neurotrophic factor (BDNF) is a major regulator of synaptoplasticity^31^ and is produced by glioblastoma^32^; LRP-4 also plays an important role in synaptic formation and function and is expressed by glioma cells as well.^33–36^

1. *IHC Analysis 1*: The significant results of univariable analyses (*P* < .1) for memory, executive functioning, and psychomotor speed are shown in Supplementary Table 7.
2. *IHC Analysis 2 (* Table 4): In multivariable analyses for all different markers, correcting for grade (WHO 2007 classification), location, and volume yielded P-STAT5b and CD163 as independent determinants for executive functioning, Semaphorin-3A for memory, and CD3 for psychomotor speed.
3. *IHC Analysis 3, multivariable analyses corrected for location and volume only* (Table 5). IDH-1, P-STAT5b, NLGN3 and CK2Beta were associated significantly with the domain executive functioning; IDH-1, BDNF, ATRX, CK2Beta, GAT-3, NLGN3, SRF, and EAAT1 with the domain memory; and IDH-1 and CD3 with the domain psychomotor speed. BDNF was associated with higher degree of cognitive impairments, the other proteins showed a protective effect on cognitive functioning.
4. *IHC analysis 4, analyses stratified for IDH-mutation (* Supplementary Table 8): In the analysis of IDH-WT gliomas, BDNF, CK2Beta and P-STAT5b were univariably associated (*P* < .1) with the domain executive functioning. For CK2Beta and P-STAT5b, these results were significant after adjusting for volume and location. For the domain memory, we found univariable associations with BDNF, Semaphorin-3A, P53 (for the IDH-mut subgroup) and SRF (for the IDH-WT group). Association between SRF and memory remained significant in multivariable analysis. ATRX was only multivariable associated with the domain memory in the IDH-mut subgroup. For the domain psychomotor speed CD3 showed significant results in the IDH-WT subgroup, both univariably and after correcting for tumor volume and location.

**Table 4:** Multivariable Analyses Corrected for Tumor Grade, Location, and Volume with Different Cognitive Domains and Thresholds as Outcome Measure

Determinant	Domain and threshold	OR (95%-CI)	*P*-value
P-STAT5b	Executive functioning SD ≤ –2	0.371 (0.143–0.703)	.005
CD163	Executive functioning SD ≤ –1	0.662 (0.443–0.988)	.044
SEMA3	Memory SD ≤ –2	3.265 (1.161–9.182)	.025
CD3	Psychomotor speed SD ≤ –2	0.509 (0.274–0.947)	.033

CI, confidence interval; OR, Odds ratio. Significant results with *p*-values

**Table 5.** Multivariable Analyses with Univariable Significant Proteins (*p* ≤ .1), Corrected for Location and Tumor-Volume

Executive functioning (SD≤-2)		
Protein	OR (95%-CI)	*P*-value
BDNF	1.365 (0.843–2.209)	.205
IDH-1	0.599 (0.183–1.960)	.397
P-STAT5b	0.303 (0.143–0.643)	.002*
CK2Beta	0.844 (0.591–1.205)	.350
EAAT1	0.970 (0.559–1.684)	.913
GAT-3	1.126 (0.706–1.797)	.619
NLGN3	0.472 (0.165–1.352)	.162
**Executive functioning (SD**≤**-1)**		
Protein	OR (95%-CI)	*P*-value
BDNF	1.374 (0.877–2.154)	.166
IDH-1	0.261 (0.097–0.701)	.008*
P-STAT5b	0.469 (0.260–0.846)	.012*
CK2Beta	0.660 (0.468–0.932)	.018*
EAAT1	0.626 (0.367–1.057)	.085
GAT-3	0.676 (0.437–1.046)	.079
NLGN3	0.399 (0.164–0.974)	.043*
SRF	0.854 (0.598–1.220)	.386
**Memory (SD**≤**-2)**		
Protein	OR (95%-CI)	*P*-value
IDH-1	0.130 (0.025–0.679)	.016*
BDNF	1.705 (1.009–2.880)	.046*
CK2Beta	0.672 (0.457–0.988)	.043*
GAT-3	0.583 (0.356–0.955)	.032*
NLGN3	0.215 (0.061–0.758)	.017*
ATRX	0.523 (0.201–1.361)	.184
EAAT1	0.714 (0.398–1.282)	.259
LRP4	0.818 (0.264–2.534)	.728
**Memory (SD**≤**-1)**		
Protein	OR (95%-CI)	*P*-value
IDH-1	0.221 (0.080–0.616)	.004*
ATRX	0.430 (0.221–0.836)	.013*
BDNF	1.668 (1.057–2.633)	.028*
CK2Beta	0.636 (0.458–0.884)	.007*
EAAT1	0.538 (0.314–0.921)	.024*
NLGN3	0.394 (0.159–0.975)	.044*
GAT-3	0.704 (0.462–1.073)	.103
LRP4	0.400 (0.145–1.101)	.076
SRF	0.575 (0.377–0.876)	.010*
**Psychomotor speed (SD**≤**-2)**		
Protein	OR (95%-CI)	*P*-value
IDH-1	0.095 (0.017–0.519)	.007*
BDNF	1.282 (0.774–2.125)	.334
CD3	0.436 (0.232–0.820)	.010*
GAT-3	0.915 (0.578–1.448)	.704
SEMAPHORIN-3A	0.154 (0.020–1.169)	.070
**Psychomotor speed (SD**≤**-1)**		
Protein	OR (95%-CI)	*P*-value
IDH-1	0.343 (0.124–0.950)	0.039*
BDNF	1.362 (0.885–2.096)	0.161
CD3	0.414 (0.221–0.776)	0.006*
GAT-3	0.830 (0.566–1.217)	0.340
NLGN3	0.460 (0.190–1.112)	0.085
SEMAPHORIN-3A	0.347 (0.113–1.071)	0.066

OR, Odds ratio; CI, confidence interval. SD = standard deviation. *=*P*-value ≤ .05

### Representability of Results

To examine the external validity of the correlations between marker expression and cognition in the awake surgery cohort, we compared expression level of all markers in the awake and non-awake surgery

cohort. Mean expression levels of IDH-1 and LRP-4 were significantly higher in the awake operated cohort, expression levels of BDNF and P-STAT5b were significantly lower in the awake cohort. All other molecular markers did not significantly differ between both groups.

## Discussion

In this cross-sectional study, we hypothesized that tumor biology contributes to an explanation for cognitive dysfunction in glioma patients. To investigate this, we examined the relation between cognitive performance (executive function, memory, and psychomotor speed) and the tumoral expression level of molecular markers in treatment-naive patients with diffuse glioma.

We found, after correcting for tumor volume and location, correlations between the expression level of CD3 or IDH-1 and psychomotor speed; IDH-1, BDNF, ATRX, CK2Beta, GAT-3, NLGN3, SRF, or EAAT1 and memory performance, and P-STAT5b, NLGN3, CK2Beta, or IDH-1 and executive functioning. After correction for histopathological grade P-STAT5b, CD163, CD3, and Semaphorin-3A expression were independently associated with cognitive deficits in different domains. After stratification for IDH-mutational status, several of these independent associations could be reproduced, particularly for P-STAT5b, CK2Beta, ATRX, SRF, and CD3.

P-STAT5b, the activated form of *signal transducer and activator of transcription 5,* is frequently activated in malignant glioma cells and has been linked to tumor development and growth.^37,38^ We also previously found that P-STAT5B associates with tumor epileptogenicity in high-grade gliomas.^39^ In this current study, upregulation of P-STAT5B correlated with a smaller change on cognitive impairments, especially for the domain executive functioning, independent of tumor grade, volume, and location. Interestingly, brain-specific STAT5 ablation is known to impair learning and memory formation in mice.^40^ Likewise, STAT5 signaling, in response to several cytokines, is required to attain normal learning and memory.^40^ P-STAT5-B can also alter the buffering and networking properties of the glial network through its regulation of the function and expression of connexins,^41,42^ and it represses the activity of the System Xc^-^ cystine-glutamate antiporter, a transporter that actively pumps cystine towards the cell in exchange for an excretion of glutamate.^43^ All these observations suggest that STAT5 activation is directly related to synaptic transmission and plasticity,^39^ possibly via a reduction of extracellular glutamate concentration and via intercellular buffering mechanisms.^20,44^ In support of this glutamatergic hypothesis, EAAT1 (*astrocytic glutamate-aspartate transporter 1)* expression correlated positively with cognitive function in our series of patients. Gliomas release large, potentially toxic concentrations of glutamate,^18^ and *EAAT1* expression could help scavenge excess extracellular glutamate around a tumor and prevent excitotoxicity.

CSNK2B encodes CK2β, the regulatory subunit of the CK2 kinase, a serine/threonine kinase associated with glioma growth and survival,^45^ but that also controls the activity and degradation of some connexins^46,47^ and the binding and extracellular secretion of fibronectin 1, a glycoprotein involved in epileptogenicity.^48^ In addition, the spectrum of interaction CK2β is broader than via CK2 alone,^49^ and mutations in this gene are responsible for some forms of congenital epilepsy,^50^ whereas CK2 inhibition has anticonvulsive and perhaps antiepileptogenic properties.^51^ CSNK2B knock down in mice decreases the formation and branching of dendrites, decreases the amplitude of miniature postsynaptic inhibitory currents^50^ and reduces the membrane expression of GLluA1 AMPA glutamate receptors.^52^

BDNF, via the activation of its receptors TrkB and p75 on neurons, modulates synaptic plasticity, neurogenesis, and neuronal survival to toxic stress. It affects in particular the activity-dependent regulation of synaptic structure and function of glutamatergic synapses, and thereby learning and memory. Deficits in BDNF expression have been associated with depression and dementia.^53^ The association of increased BDNF expression with worse cognitive performance in our patients is thus unlikely causal, albeit BDNF can also modify glutamate signaling directly, by changing the expression of glutamate receptor subunits and Ca2+-regulating proteins in neurons, and directly activate post-synaptic NMDA receptors and could thus further offset the interplay between neurotransmitters in the vicinity of gliomas. Another explanation to our finding could be that BDNF expression in the tumor is a mere surrogate marker of the pathological glutamate secretion by tumor cells, since glutamate stimulates the production of BDNF by surrounding neurons, glial cells.^54^

Interestingly, the expression of GAT-3, the high-affinity transporter for the inhibitory neurotransmitter, GABA, associated with increased memory performance in our patients. GAT-3 is normally expressed by astrocytes,^55^ and removes this amino-acid from the synaptic microenvironment. Our findings thus suggest beside glutamatergic signaling, the deregulation of other neurotransmitter concentrations in the vicinity of gliomas can cause cognitive alterations that are independent of tumor size and location.

Semaphorins are a family of secreted and (trans)membrane glycosylphosphatidylinositol-linked glycoproteins that guide axon growth in the developing neural system and later control synaptogenesis, axon pruning, the density and maturation of dendritic spines, synaptic physiology, and neuronal excitability.^56^ They act by their plexin receptors and neuropilin co-receptors, and semaphorin 3 and its (co-)receptors are expressed by glioma cells.^57^ Semaphorins can promote excitatory glutamatergic synapse density and function in the hippocampus,^58^ and in our study, indeed, Semaphorin-3A had a protective effect on the domain memory, even after correction for grade and other confounders. That Semaphorins can have a positive effect on memory without altering other cognition domains like other regulators of the glutamatergic neurotransmission can result from the context-dependent actions of semaphorins and their receptors, as well as frorm its other globally positive effects on the anatomy of synapses. At any rate, this suggests that multiple metabolic mechanisms contribute and compete to determine the cognitive effects of glial tumors, independent of the size, grade, and location of the tumors.

Neuroligin-3 (NLGN3), a postsynaptic cell-adhesion protein, was recently identified as a key factor in the interaction between the brain’s neurons and glioma cells. Several groups showed that a direct synaptic communication between neurons and glioma cells exists with potential clinical implications, such as epileptic seizures. NLGN3 is a key component in this mode of communicating. It is secreted through neuronal activity and induces its own expression in tumor cells.^31,35,36,59^ Increased excitatory neuronal activity, in turn, significantly enhances tumor growth. NLGN3 is strongly negatively correlated with patients’ overall survival.^35,36^ In physiologic situations NLGN3 is essential for synaptic function, and is present in both excitatory and inhibitory synapses.^60^ The family of neurexins and neuroligins shape synaptic plasticity and efficacy, and may influence cognitive functioning. In our data, we found a significant and independent protective effect of NLGN3 on memory and executive functioning, independent of tumor volume and location. Speculatively, the effects of NLGN3 on both excitatory and inhibitory synapses—and consequently on neural networks—could both promote tumor growth and help preserve cognition. Further studies on the relationship between NLGN3 and NCF may include measures for functional connectivity, eg with resting-state functional MRI or magnetoencephalography.

CD163 and CD3 are specific markers of T-lymphocytes and myeloid/microglial cells. In our study, we observed that the higher the tumor infiltration by these cells, the better the cognition, especially in the domain of cognitive speed. This is rather counter intuitive, as the expression of pro-inflammatory cytokines in macrophage-like cells and microglia cells in the brain, as in case of local or general infection for instance, is responsible of lethargy and decreased social behavior.^61^ Likewise, microglia mediate inflammation, neuronal death, and aberrant neurogenesis after epileptic seizures.^62^ T-lymphocytes and microglial cells can however influence synaptic transmission in positive ways as well as microglial cells can also exert inflammation-independent functions and contribute to both the formation and the pruning of neuronal synapses,^63^ and T-cell release of IL-1 can favor synaptic strengthening (long term potentiation, or LTP).^64^ These mechanisms could help scavenge or repair dysfunctional synapses in the vicinity of tumor cells.

Of note, IDH-1 and ATRX mutations are also associated with cognitive deficits. IDH mutations result in the generation of 2-hydroxyglutarate and have been found to associate favorably with cognition previously.**^21^** ATRX mutations on the other hand result in a loss of function of this chromatin binding protein, essential in the proper function of the telomere, but are not known to result in metabolic changes.^65^ Both IDH and ATRX mutations are however hallmarks of tumors of lower grade.^15,66^ The fact that their association with cognitive performance does not remain significant after correction for histological grade may simply reflect that tumors that harbor these mutations simply grow slower than those that do not, rather than metabolic changes in the tumor microenvironment.

Our study results must be interpreted in the light of some limitations. First, selection bias might have played a role, because only patients undergoing awake surgery were included in our study sample. For this reason we compared baseline tumor- and patient-related characteristics of the included patients with patients who underwent glioma surgery under general anesthesia in the same period of time in former studies.^6^ Results of this comparison showed that the awake operated patients are a specific group of patients, with relatively good clinical performance and they are almost always selected based on the eloquent localization of the tumor. However, we included a large number of right-sided tumors, which differs from selection criteria for awake surgery in other centers. As such, the cohort represents a rather broad selection of all diffuse glioma patients, despite the fact that we only included awake surgery patients. Of note, patients who are selected for awake operation generally are not too severely affected; this selection process probably caused an underestimation of the cognitive problems in the complete spectrum of glioma patients at the population level.

In addition, patients with available fresh frozen tissue for GSEA analyses (step 1) were mainly males (74%) and the percentage of grade II and III was relatively high (Supplementary Table 5). This may have biased the results of GSEA analyses, since grade may influence cognitive functioning, and probably correlates with expression of certain molecular markers. However, we corrected for grade in our differential expression analyses and results did not point in the direction of IDH-driven pathways, meaning that we probably corrected for grade sufficiently. The influence of tumor-degree on our IHC analyses was tested in subgroups. Indeed, some proteins appeared to interact with grading or IDH-mutation as their associations with cognition did not remain significant in IDH-stratified analyses. However, this could also be the result of the smaller sample size in subgroups. In addition, some proteins maintained a significant relationship with cognitive domains in these subgroups, such as STAT5b, CD3, and CK2Beta. For these proteins, associations with cognition cannot be explained or driven by IDH-mutation or grading of the tumor. For other protein markers, it may be that their relation with cognition is influenced by the (genetic, microenvironmental, and other) differences between IDH-mutated and IDH-wildtype gliomas.

Another possible source of bias is the selective loss of patients who had insufficient neuropsychological data to perform analyses on. The reason for having insufficient data was often emergency surgery in case of rapid clinical decline, so this could also have led to an underestimation of neurocognitive problems and, possibly, missing other significant metabolic/cognitive associations.

Furthermore, possible interference by common pitfalls of immunohistochemistry must be considered. Single-observer assessment, sampling error (given the strong tumor-heterogeneity in glioma), and a semi-quantitative scoring approach (a score of 3 covered a broad range of immunoreactivity: 50–100% positive cells) on triplicate tumor cores could have hampered accuracy in obtaining protein expression data. As consequence of the latter, majority of core samples were also given a score of 3, which limited discriminative as well as statistical power (for the lower expression subgroups). Accuracy might have also been affected by missing of core samples in the triplicates, as less samples of the heterogenic tumor were represented in the mean expression scores in those cases. This might have caused over- or underestimated mean scores for some individual patients. Significant impact on group-level could have occurred if missing data were not at random. To minimize this source of bias, we discussed samples with missing scores with a neuropathologist (WvH).

Finally, we found a low number of significantly associated gene sets in our GSEA analysis. The choice of markers for our second phase of analysis was partly based on previous knowledge and literature and therefore our results have to be considered as explorative. It is possible that the low number of associations we found with our GSEA analysis reflects a power problem in the analysis of the relationship between neurocognitive assessments and gene expression. On the other hand, it is also possible that the associations we *did* find in this study are the result of chance due to the low power, for this reason they need to be validated in a larger cohort.

Strengths of this study include the large sample size, when compared to the few previous studies in this field, and the extensiveness of data on pre-operative NCF. All neuropsychological data were prospectively collected and registered, and tested according to a standard clinical procedure, leading to a homogeneous set of neuropsychological tasks. The two-step approach, with hypothesis-free identification of markers in GSEA, and validation (of GSEA findings and previously reported markers) in the IHC analysis, allowed for an efficient use of available data and added to robustness of our results.

Our findings suggests that variations in glioma biology influence cognitive performance through mechanisms that include perturbation of neuronal and non-neuronal signaling. These findings can improve understanding of the pathophysiology underlying cognitive deficits in glioma. If the mechanisms and pathways underlying our findings can be unraveled in future studies, this could facilitate clinical risk stratification for development of cognitive sequelae among glioma patients. For reliable recommendations regarding these correlations and their potential therapeutic implications, we strongly advocate validation of our findings in a prospectively designed external cohort with more targeted IHC techniques and further correction for possible clinical covariates.

## Conclusion

Our results suggest that glutamatergic signaling, possibly infiltrating immune cells, contribute to neurocognitive dysfunction in glioma patients, independent of tumor size, grade, and location. Upon confirmation, these results can pave the way to therapeutic trials aiming to prevent or restore cognitive deterioration in these patients.

## Supplementary Material

noac036_suppl_Supplementary_Table_S1

noac036_suppl_Supplementary_Table_S2

noac036_suppl_Supplementary_Table_S3

noac036_suppl_Supplementary_Table_S4

noac036_suppl_Supplementary_Table_S5

noac036_suppl_Supplementary_Table_S6

noac036_suppl_Supplementary_Table_S7

noac036_suppl_Supplementary_Table_S8

noac036_suppl_Supplementary_Figure_S1

noac036_suppl_Supplementary_Figure_Legend
