# Supplementary material for: Tumor-related molecular determinants of neurocognitive deficits in patients with diffuse glioma
Source: Neuro Oncol. 2022 Feb 11;24(10):1660–70. doi: 10.1093/neuonc/noac036 (PMC9527514; doi:10.1093/neuonc/noac036)
Supplement: noac036_suppl_Supplementary_Table_S1 [file noac036_suppl_supplementary_table_s1.docx]

**Supplementary table 1: Neuropsychological tasks per domain**

| Attention & Executive Functioning |
| --- |
| Wechsler Adult Intelligence Scale (WAIS-III) Digit Span Forward^a^  Trail Making Test (TMT) Switching ratio (TMTB/TMTA)^b^  Phonologic Fluency^c^  Stroop/Delis Kaplan Executive Function System (DKEFS) inhibition ratio^d^ |
| Memory |
| Wechsler Adult Intelligence Scale (WAIS-III) Digit SpanBackward  RAVLT-Dutch Version immediate, delay, recognition^e^  Rey-Osterieth Complex Figure Test (ROCF) delay^f^  Semantic Fluency^g^ |
| Psychomotor Speed |
| Stroop/DKEFS I  Stroop/DKEFS II  TMTA |

1. Wechsler Adult Intelligence Scale Third Edition Digit Span [WAIS-III] (WAIS-III Administration and scoring manual, 1997), Wechsler Adult Intelligence Scale Fourth Edition Digit Span [WAIS-IV] (WAIS-IV-NL Technische handleiding, 2013)
2. Trail Making Test [TMT] (Giovagnoli, Del Pesce, Mascheroni, Simoncelli, Laiacona, & Capitani, 1996)
3. Phonologic Verbal Fluency Test [Lexical Fluency] (Harrison, Buxton, Husain, & Wise, 2010; Schmand, Groenink, & Van Den Dungen, 2008)
4. The Stroop Color and Word Test [Stroop] (MacLeod, 1991), Color Word Interference Test (Benton, Sivan, Hamsher, Varney, & Spreen, 1994)
5. 15 Words Test [15WT] (Saan & Deelman, 1986)
6. Rey-Osterieth Complex Figure Test [ROCF] (Berry & Carpenter, 1992; Spreen & Strauss, 1998)
7. Semantic Verbal Fluency Test [Semantic Fluency] (Harrison et al., 2010)
