# Supplementary material for: Tumor-related molecular determinants of neurocognitive deficits in patients with diffuse glioma
Source: Neuro Oncol. 2022 Feb 11;24(10):1660–70. doi: 10.1093/neuonc/noac036 (PMC9527514; doi:10.1093/neuonc/noac036)
Supplement: noac036_suppl_Supplementary_Table_S2 [file noac036_suppl_supplementary_table_s2.docx]

**Supplementary table 2: Antibodies and dilution used for immunohistochemistry**

| Target | Antibody and dilution |
| --- | --- |
| p-STAT5B | anti-STAT5b (phospho S731) (Rabbit polyclonal, dilution 1:200, Abcam, citrate buffer) |
| H3 | anti-histone H3 (acetyl K9) (Rabbit polyclonal, dilution 1:800, Abcam, citrate buffer) |
| p-ROCK1 | ROCK1 (phospho T455 + S456) (Rabbit polyclonal, dilution 1:200, Abcam, EDTA buffer) |
| BDNF | BDNF (Rabbit monoclonal, dilution 1:1000, Abcam, citrate buffer) |
| LRP-4 | LRP4 (Rabbit polyclonal, dilution 1:50, Abcam, EDTA buffer) |
| Connexin 30 | Connexin 30 (Z-PP9) (Rabbit polyclonal, dilution 1:500, ThermoScientific, citrate buffer) |
| CD3 | CD003 (Rabbit monoclonal, dilution 1:100, Abcam, EDTA buffer) |
| SRF1  SRF2 | SRF 1 anti-SRF (Rabbit polyclonal, dilution 1:100, Abcam, EDTA-buffer)  SRF2 anti-SRF (Rabbit polyclonal, dilution  1:50, SIGMA, citrate buffer) |
| STAT3 | Stat3 (phospho Tyr705) (Rabbit monoclonal, dilution 1:100, CellSignaling, citrate buffer) |
| CD163 | CD163 (Mouse monoclonal, dilution 1:800, Abcam, EDTA-buffer) |
| IDH-1 | IDH1 (Mouse monoclonal, dilution 1:80, DIANOVA, EDTA-buffer) |
| ATRX | ATRX (Rabbit polyclonal, dilution 1:300, SIGMA, EDTA-buffer) |
| P53 | P53 (Rabbit monoclonal, dilution 1:100, ROCHE, EDTA-buffer) |
| EAAT1 | Anti-EAAT1 antibody (Rabbit monoclonal, dilution 1:2000, Abcam, citrate buffer) |
| EEAT2 | EAAT 2 (Rabbit monoclonal, dilution 1:150, Abcam, citrate buffer) |
| GAT-3 | GABA (Rabbit, polyclonal, dilution 1:100, Abcam, citrate buffer) |
| CK2Beta | Anti-Casein Kinase 2 beta antibody (Rabbit monoclonal, Dilution 1:50, Abcam, citrate buffer) |
| Semaphorin-3A | Anti-Semaphorin-3A antibody (Rabbit polyclonal, dilution 1:100, Abcam, EDTA-buffer)  Plexin |
| Plexin A1 | Plexin A1 antibody (Rabbit polyclonal, dilution 1 :200, Novus biologicals, citrate buffer) |
