# Supplementary material for: Tumor-related molecular determinants of neurocognitive deficits in patients with diffuse glioma
Source: Neuro Oncol. 2022 Feb 11;24(10):1660–70. doi: 10.1093/neuonc/noac036 (PMC9527514; doi:10.1093/neuonc/noac036)
Supplement: noac036_suppl_Supplementary_Table_S3 [file noac036_suppl_supplementary_table_s3.docx]

**Supplementary table 3: Materials and methods**

*Patient selection and study design*

A cross-sectional study of a consecutive cohort of 793 adult patients with proven diffuse glioma (WHO grade II-IV) at the UMCU was conducted. The participants all underwent first surgical tumor resection under either awake conditions (n=197) or general anesthesia (n=596) in the period of January 2010 to January 2017. All awake craniotomy patients underwent elaborate neuropsychological testing as part of their pre-operative work-up while patients operated under general anesthesia did not. So, for the ‘awake surgery’ group, neuropsychological testing data were prospectively obtained by clinical neuropsychologists (within one week prior to surgery). The ‘awake surgery’ group was used to investigate the correlation between protein expression levels and cognitive performance. Executive functioning, memory and psychomotor speed were the cognitive domains chosen as primary outcome measures, because in earlier studies these domains were found to be most vulnerable^1^. The non-awake operated group served as control group to test both technical validity of the analysis in the ‘awake surgery’ group and the external representability of their biomolecular tumor profile to all glioma patients in general.

*Baseline characteristics and neuropsychological tests*

Patient characteristics were retrospectively retrieved from the electronic patient files. Those included age, gender, Karnofsky Performance Score (KPS), WHO 2016 classification of gliomas, and tumor localization and volume. For each patient the pre-operative MRI (both T2/fluid attenuation inversion recovery (FLAIR) and T1 after gadolinium administration) was reviewed and data were acquired by a junior clinical scientist (EvK) under supervision of a senior neuro-oncologist (TJS). Involvement of the following structures was determined visually for both hemispheres; frontal, parietal, temporal, occipital, hippocampus, insular and multifocal involvement. Volumes were measured in 3D with use of Osirix Lite (v. 9.5.2) on T2-/FLAIR-weighted MRI scans and the volume was defined as the whole area of hyperintensity. This represents the total lesion volume, including tumor and edema. Volumes were measured by a junior clinical scientist (EvK).

The neuropsychological instruments that were used as part of our preoperative routine clinical care are listed in supplementary table 1. Neuropsychological tests often tap more than one cognitive domain. We used a predetermined test classification (supplementary table 1). Despite a standard test battery, the testing was, in line with clinical care, tailored to the individual patient’s cognitive complaints, location of the tumor and medical condition.

*Step 1: mRNA Expression Analysis and pathway analysis*

Sixty-five fresh-frozen surgical samples of de novo diffuse gliomas were prospectively collected between 2010 and 2015. RNA was extracted with the Nucleospin® TriPrep (Macherey-Nagel, Düren, Germany) and the QIASymphony RNA (Qiagen, Venlo, The Netherlands) kits according to the manufacturers’ instructions. Affymetrix HG U133 plus 2.0 arrays were prepared and scanned according to the manufacturer’s protocol and as reported previously ^2^. Quality control and differential gene expression analyses were performed with RStudio (v1.1.463). Robust Multi-array Average (RMA) normalization was applied. We analyzed differential expression with the ‘limma’ package with correction for batch, gender and age. Gene set enrichment analyses (GSEA’s) were performed with software provided from the broad institute as reported previously ^3^. The following parameters were used; 1000 permutations, FDR cut off ≦ 0.25, chip platform file; Human_AFFY_HG_U133_MSigDB.v7.0.chip, gene sets database c1.all.v7.1.symbols.gmt – c7.all.v7.1.symbols.gmt.

## *Step 2: Tissue microarray and Immunohistochemistry*

TMA construction and immunostaining were performed at the pathology department of the UMCU. For each patient, three representative tumor zones for core sampling were delineated and marked on the original haematoxylin and eosin (HE)-stained samples by two neuropathologists (WvH and WGMS). TMA’s - identical to corresponding predesigned TMA templates - were subsequently prepared, in accordance with standard protocols. Cylindrical tissue core biopsies of the marked tumor areas were taken from the donor paraffin blocks with use of a manual arrayer (Beecher Instruments) and re-embedded in empty deparaffinated recipient paraffin blocks at the designated coordinates. Each block also contained three core samples for orientation (kidney, liver or pancreas tissue). With use of a microtome, 4 μm sections were cut from the blocks, resulting in 13 TMA slides (sorted by tumor grade and phenotype) for each molecular marker.

Protein expression assessment was blinded to clinical data and performed by means of light microscopy (magnifications 25x, 50x, 100x, 200x). The tumor samples were scored by medical students (HV, EAK, AEB) and junior clinical scientists (EvK, SB), who all underwent training by an experienced neuropathologist (WvH and WGMS). Samples were reviewed by WvH or WGMS in case of doubt. Protein expression was quantified by means of number of positive tumor cells (cytoplasmic and/or nuclear positivity)/field and converted to a score on the following ordinal scale: 0 (<5% immunoreactive cells), 1 (5-25%), 2 (25-50%), 3 (50-100%). The final protein expression score of each patient was represented by the mean score of all (one, two or three) evaluated core samples.

References

**1.** van Kessel E, Emons MAC, Wajer IH, et al. Tumor-related neurocognitive dysfunction in patients with diffuse glioma: a retrospective cohort study prior to antitumor treatment. *Neuro-Oncology Practice.* 2019.

**2.** Turkheimer FE, Roncaroli F, Hennuy B, et al. Chromosomal patterns of gene expression from microarray data: methodology, validation and clinical relevance in gliomas. *BMC Bioinformatics.* 2006; 7:526.

**3.** Subramanian A, Tamayo P, Mootha VK, et al. Gene set enrichment analysis: a knowledge-based approach for interpreting genome-wide expression profiles. *Proc Natl Acad Sci U S A.* 2005; 102(43):15545-15550.
