# Supplementary material for: Tumor-related molecular determinants of neurocognitive deficits in patients with diffuse glioma
Source: Neuro Oncol. 2022 Feb 11;24(10):1660–70. doi: 10.1093/neuonc/noac036 (PMC9527514; doi:10.1093/neuonc/noac036)
Supplement: noac036_suppl_Supplementary_Table_S4 [file noac036_suppl_supplementary_table_s4.docx]

**Supplementary table 4: Different types of univariable and multivariable analyses for immunohistochemistry data**

| Analysis number | Type of analyses, determinants and outcome measure | Results printed in |
| --- | --- | --- |
| 1 | Univariable analyses for all different proteins. For three domains for both thresholds | Supplementary table 6 (only results with p<0.1) |
| 2 | Multivariable analyses corrected for grade, location and volume for all different proteins. For three domains for both thresholds | Table 4 |
| 3 | Multivariable analyses corrected for location and volume, selection of proteins based on the univariable analyses (1). For three domains for both thresholds | Table 5 |
| 4 | Analyses (1) and (3) stratified by IDH-mutation | Supplementary table 7 |
