# Supplementary material for: Tumor-related molecular determinants of neurocognitive deficits in patients with diffuse glioma
Source: Neuro Oncol. 2022 Feb 11;24(10):1660–70. doi: 10.1093/neuonc/noac036 (PMC9527514; doi:10.1093/neuonc/noac036)
Supplement: noac036_suppl_Supplementary_Table_S5 [file noac036_suppl_supplementary_table_s5.docx]

|  | N (%)/range |
| --- | --- |
| Overall | 65 |
| Sex (Female) | 17 (26.2) |
| Age at first surgery (mean (SD)) | 50 (23-82) |
| Domain Memory impaired | 11 (19.3) |
| Domain Executive functioning impaired | 14 (24.6) |
| Karnofsky Performance Score (median [IQR]) | 70 (30-100) |
| Volume (cm^3)^ (median [IQR]) | 26.72 (1.5-51) |
| WHO 2007 classification | |
| Grade II | 23 (40.4) |
| Grade III | 13 (20.0) |
| Grade IV | 21 (36.8) |
| Location (measured on T2 FLAIR)^*^  Left frontal  Left parietal  Left temporal  Left occipital  Right frontal  Right parietal  Right temporal  Right occipital | 32 (57.1)  13 (23.2)  19 (33.9)  7 (12.5)  8 (14.3)  2 (3.6)  5 (7.7)  1 (1.5) |

**Supplementary table 5: Baseline characteristics of all patients with available fresh frozen tissue, included for GSEA analysis**

*Percentages add up above 100% due to involvement of multiple lobes in each patient
