# Supplementary material for: Tumor-related molecular determinants of neurocognitive deficits in patients with diffuse glioma
Source: Neuro Oncol. 2022 Feb 11;24(10):1660–70. doi: 10.1093/neuonc/noac036 (PMC9527514; doi:10.1093/neuonc/noac036)
Supplement: noac036_suppl_Supplementary_Table_S6 [file noac036_suppl_supplementary_table_s6.docx]

**Supplementary table 6:** Overall location of all eligible patients, who underwent awake surgery between 2010 and 2017

| Location | N (% of total n=197) |
| --- | --- |
| L frontal | 21 (10.7) |
| L temporal +/- occipital | 12 (6.1) |
| L parietal +/- occipital | 9 (4.6) |
| L frontoinsular | 15 (7.6) |
| L temporoinsular | 11 (5.6) |
| L frontotemporal +/- insula | 15 (7.6) |
| L frontoparietal +/- insula, +/- occipital | 13 (6.6) |
| L temporoparietal +/- insula, +/- occipital | 9 (4.6) |
| L frontotemporoparietal +/- insula +/- occipital | 14 (7.1) |
| Bifrontal | 8 (4.1) |
| R frontal | 7 (3.6) |
| R temporal +/- occipital | 2 (1.0) |
| R parietal +/- insula, +/- occipital | 3 (1.5) |
| R frontoinsular | 4 (2.0) |
| R temporoinsular | 1 (0.51) |
| R frontotemporal +/- insula | 8 (4.1) |
| R frontoparietal +/- insula, +/- occipital | 12 (6.1) |
| R temporoparietal +/- insula, +/- occipital | 1 (0.51) |
| R frontotemporoparietal +/- insula +/- occipital | 8 (4.1) |

Location in this table was based on the, visually determined, involvement of the tumor in the different brain lobes. Any degree of T2-hyperintensity in a lobe (however small) was considered as lobe involvement. Basal ganglia, hippocampus and brainstem/cerebellum were not considered separately in this classification'
