# Supplementary material for: Tumor-related molecular determinants of neurocognitive deficits in patients with diffuse glioma
Source: Neuro Oncol. 2022 Feb 11;24(10):1660–70. doi: 10.1093/neuonc/noac036 (PMC9527514; doi:10.1093/neuonc/noac036)
Supplement: noac036_suppl_Supplementary_Table_S7 [file noac036_suppl_supplementary_table_s7.docx]

**Supplementary table 7: Univariable analyses for association between molecular markers and different cognitive domains.** Results with p-values ≤0.1 are shown. *=p-value ≤0.05

**Executive functioning (SD**≤**2)**

| Protein | OR (95% CI) | p-value |
| --- | --- | --- |
| BDNF | 1.764 (1.204-2.585) | 0.004* |
| IDH-1 | 0.332 (0.126-0.875) | 0.026* |
| P-STAT5B | 0.554 (0.318-0.966) | 0.037* |
| ATRX | 0.507 (0.244-1.052) | 0.068 |

**Executive functioning (SD**≤**-1)**

| Protein | OR (95% CI) | p-value |
| --- | --- | --- |
| BDNF | 1.668 (1.136-2.449) | 0.009* |
| IDH-1 | 0.252 (0.107-0.594) | 0.002* |
| CK2Beta | 0.641 (0.465-0.883) | 0.007* |
| EAAT1 | 0.594 (0.361-0.979) | 0.041* |
| GAT-3 | 0.647 (0.449-0.934) | 0.020* |
| NLGN3 | 0.396 (0.174 – 0.903 | 0.028 |

**Memory (SD**≤-**2)**

| Protein | OR (95% CI) | p-value |
| --- | --- | --- |
| IDH-1 | 0.103 (0.022-0.470) | 0.030* |
| BDNF | 2.145 (1.390-3.310) | 0.001* |
| CK2Beta | 0.713 (0.521-0.977) | 0.035* |
| GAT-3 | 0.629 (0.429-0.920) | 0.017* |
| P53 | 1.447 (0.930-2.252) | 0.101 |
| NLGN3 | 0.258 (0.087 – 0.770) | 0.015 |

**Memory (SD**≤**-1)**

| Protein | OR (95% CI) | p-value |
| --- | --- | --- |
| IDH-1 | 0.179 (0.075-0.430) | <0.001* |
| ATRX | 0.429 (0.239-0.769) | 0.005* |
| BDNF | 1.895 (1.282 – 2.801) | 0.001* |
| CK2Beta | 0.643 (0.474-0.872) | 0.005* |
| EAAT1 | 0.531 (0.319-0.884) | 0.015* |
| GAT-3 | 0.686 (0.484-0.973) | 0.034* |
| SRF | 0.648 (0.458-0.916) | 0.014* |
| LRP-4 | 0.453 (0.180-1.141) | 0.093 |
| NLGN3 | 0.397 (0.175 – 0.899) | 0.027 |

**Psychomotor speed (SD**≤**-2)**

| Protein | OR (95% CI) | p-value |
| --- | --- | --- |
| IDH-1 | 0.086 (0.019-0.393) | 0.002* |
| BDNF | 1.607 (1.08-2.389) | 0.019* |
| CD3 | 0.555 (0.342-0.902) | 0.017* |
| Semaphorin-3A | 0.135 (0.019-0.967) | 0.046* |
| ATRX | 0.452 (0.196-1.041) | 0.062 |
| EAAT1 | 0.666 (0.434-1.024) | 0.064 |

**Psychomotor speed (SD**≤**-1)**

| Protein | OR (95% CI) | p-value |
| --- | --- | --- |
| IDH-1 | 0.250 (0.099-0.634) | 0.003* |
| BDNF | 1.491 (1.045-2.127) | 0.028* |
| CD3 | 0.505 (0.300-0.849) | 0.010* |
| Semaphorin-3A | 0.319 (0.102-0.994) | 0.049* |
| GAT-3 | 0.739 (0.529-1.031) | 0.075 |
