# Supplementary material for: Tumor-related molecular determinants of neurocognitive deficits in patients with diffuse glioma
Source: Neuro Oncol. 2022 Feb 11;24(10):1660–70. doi: 10.1093/neuonc/noac036 (PMC9527514; doi:10.1093/neuonc/noac036)
Supplement: noac036_suppl_Supplementary_Table_S8 [file noac036_suppl_supplementary_table_s8.docx]

**Supplementary table 8: Significant results of subgroup analyses stratified by IDH-mutation.** CI = confidence interval. *=p-value ≤0.05. Multivariable analyses are corrected for location and tumor volume. IDHWT=IDH-wildtype; IDH-mut = IDH-mutant; OR=odds ratio; 95%-CI = 95%-confidence interval. Multivariable analyses in this table are based on the results of univariable analyses of the whole (non-stratified) study sample.

| Univariable analyses | | | | | |
| --- | --- | --- | --- | --- | --- |
| Protein | **OR** | **95% CI** | **p-value** | **Subgroup** | **Domain** |
| BDNF | 1.704 | 0.907-3.201 | 0.098 | IDHWT | Executive functioning SD ≤ -1 |
| CK2Beta | 0.472 | 0.249-0.894 | 0.021* | IDHWT | Executive functioning SD ≤ -1 |
| P-STAT5b | 0.529 | (0.268-1.044) | 0.066 | IDHWT | Executive functioning SD ≤ -2 |
| BDNF | 3.333 | 1.074-10.340 | 0.037 | IDH-mut | Memory SD ≤ -2 |
| SEMA-3A | 2.310 | 0.950-5.613 | 0.065 | IDH-mut | Memory SD ≤ -2 |
| P53 | 4.743 | 0.882-25.493 | 0.070 | IDH-mut | Memory SD ≤ -2 |
| SRF | 0.297 | 0.079-1.116 | 0.072 | IDHWT | Memory SD ≤ -1 |
| CD3 | 0.573 | 0.297-1.107 | 0.097 | IDHWT | Psychomotor Speed SD ≤ -1 |
| Multivariable analyses | | | | | |
| P-STAT5b | 0.181 | (0.047-0.695) | 0.013* | IDHWT | Executive functioning SD ≤ -2 |
| CK2Beta | 0.396 | (0.195-0.805) | 0.011* | IDHWT | Executive functioning SD ≤ -1 |
| ATRX | 0.408 | 0.158-0.9951.051 | 0.04963 | gr. II/III IDH-mut | Memory SD ≤ -1 |
| SRF | 0.228 | (0.044-1.177) | 0.077 | IDHWT | Memory SD ≤ -1 |
| CD3 | 0.380 | 0.156-0.926 | 0.033* | IDHWT | Psychomotor Speed SD ≤ -1 |
