# Supplementary figures and images for: Tumor-related molecular determinants of neurocognitive deficits in patients with diffuse glioma
Source: Neuro Oncol. 2022 Feb 11;24(10):1660–70. doi: 10.1093/neuonc/noac036 (PMC9527514; doi:10.1093/neuonc/noac036)

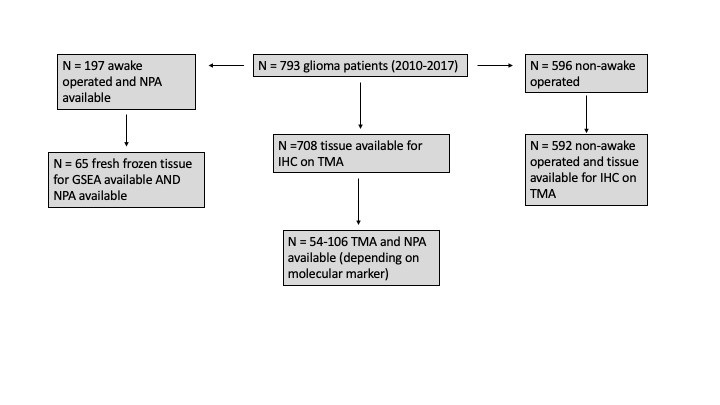

Supplement: noac036_suppl_Supplementary_Figure_S1 [file noac036_suppl_supplementary_figure_s1.jpeg]
