# Supplementary material for: Tumor-related molecular determinants of neurocognitive deficits in patients with diffuse glioma
Source: Neuro Oncol. 2022 Feb 11;24(10):1660–70. doi: 10.1093/neuonc/noac036 (PMC9527514; doi:10.1093/neuonc/noac036)
Supplement: noac036_suppl_Supplementary_Figure_Legend [file noac036_suppl_supplementary_figure_legend.docx]

**Figure captions**

Fig. S1. Flowchart of the included patients. TMA= Tissue Microarray. NPA = Neuropsychological Assessment. GSEA = Gene Set Enrichment Analysis. IHC = Immunohistochemistry
